# Formal and Informal Caregiving: Implications for Paid Family Caregivers Under the Consumer-Directed Model

**DOI:** 10.1093/geroni/igaa057.1897

**Published:** 2020-12-16

**Authors:** Naomi Gallopyn, Kathrin Boerner, Lisa Iezzoni

**Affiliations:** 1 Massachusetts General Hospital, Boston, Massachusetts, United States; 2 University of Massachusetts Boston, Boston, Massachusetts, United States

## Abstract

The case of paid family caregiving under the consumer-directed model introduces unique complexities to patient care, especially in the presence of other formal caregiving. The purpose of this study was to address the interface of paid and family caregiving by exploring scenarios where the two types of caregiving overlap or are merged into one role. Participants were 20 formal caregivers and 21 persons with significant disability, recruited with a purposive sampling approach. In-depth, qualitative interviews were conducted with caregivers and patients to triangulate information from different perspectives. Thematic analysis uncovered pathways into formal caregiving that involved a history of family caregiving, transitions from informal family caregiving to formal family caregiving under the consumer-directed model, and challenges that emerged when formal and informal caregivers co-exist in the same environment. Implications for patient and caregiver experiences in care contexts where the boundaries between paid and unpaid care are blurred are discussed.

